# Progress in Mesoporous Silica Nanoparticles as Drug Delivery Agents for Cancer Treatment

**DOI:** 10.3390/pharmaceutics13020152

**Published:** 2021-01-24

**Authors:** Eleen Dayana Mohamed Isa, Haslina Ahmad, Mohd Basyaruddin Abdul Rahman, Martin R. Gill

**Affiliations:** 1 Department of Chemical and Environmental Engineering, Malaysia-Japan International Institute of Technology, Universiti Teknologi Malaysia, Jalan Sultan Yahya Petra, Kuala Lumpur 54100, Malaysia; eleen92@gmail.com; 2 Department of Chemistry, Faculty of Science, Universiti Putra Malaysia, UPM Serdang 43000, Malaysia; basya@upm.edu.my; 3 UPM-MAKNA Cancer Research Laboratory, Institute of Bioscience, Universiti Putra Malaysia, UPM Serdang 43400, Malaysia; 4 Department of Chemistry, Swansea University, Swansea SA2 8PP, UK; m.r.gill@swansea.ac.uk

**Keywords:** mesoporous silica nanoparticles, drug delivery, cancer

## Abstract

Cancer treatment and therapy have made significant leaps and bounds in these past decades. However, there are still cases where surgical removal is impossible, metastases are challenging, and chemotherapy and radiotherapy pose severe side effects. Therefore, a need to find more effective and specific treatments still exists. One way is through the utilization of drug delivery agents (DDA) based on nanomaterials. In 2001, mesoporous silica nanoparticles (MSNs) were first used as DDA and have gained considerable attention in this field. The popularity of MSNs is due to their unique properties such as tunable particle and pore size, high surface area and pore volume, easy functionalization and surface modification, high stability and their capability to efficiently entrap cargo molecules. This review describes the latest advancement of MSNs as DDA for cancer treatment. We focus on the fabrication of MSNs, the challenges in DDA development and how MSNs address the problems through the development of smart DDA using MSNs. Besides that, MSNs have also been applied as a multifunctional DDA where they can serve in both the diagnostic and treatment of cancer. Overall, we argue MSNs provide a bright future for both the diagnosis and treatment of cancer.

## 1. Introduction

Cancer remains a challenge and leading cause of death. Based on GLOBALCON, it was estimated at approximately 18.1 million new cancer cases and 9.6 million mortality due to cancer in 2018 [1]. The most commonly diagnosed cancer for each gender was found to be lung cancer, and it is also the leading cause of death by cancer. Other types of cancer commonly diagnosed are female breast cancer, prostate cancer and colorectal [1]. Up to the current date, the most widely used treatment for these patients is chemotherapy, where the drugs are intravenously injected into the body. However, the main apprehension with these drugs is their adverse side effects [2]. Therefore, the main priorities in cancer research are drug discovery with higher efficacy against cancer cells (targeted therapeutics) or to develop and improve the drug delivery systems, which then increase the drug effectiveness against cancerous cells.

In the effort of developing a drug delivery system, nanotechnology has been explored as one of the main platforms in the last few decades. Nanomaterials can be generally defined as materials with a size ranging 1–100 nanometers. However, in the field of medicine, nanomaterials, which are being used as a drug delivery agent commonly referred to as nanomedicine, size typically reaches up to several hundred nanometers [3]. The first developed nanomedicine was reported in the early 1960s where liposomes served as the carrier. Since then, various carriers have been developed to consistently improved to increase the effectiveness of therapeutic delivery [4]. Nanoparticles are like a blank canvas, and the main advantages in the development of nanomedicine are it able to increase drug bioavailability, better interaction with the biological system within the same size, high surface area and functionality, the possibility of changing its physical properties and the possibility of a multifunctional system with different properties [5]. In general, these nanoparticles can be classified into two categories, which are organic and inorganic. Inorganic nanoparticles pose a few advantages over organic nanoparticles in terms of stability [6]. One of the inorganic materials that are popular in the development of drug delivery agents for anticancer treatment is mesoporous silica nanoparticles (MSNs).

MSNs can be defined as silica materials within the nanometer range that contain porosity. According to IUPAC classification, MSNs’ pores diameter is within 2 to 50 nm [7]. It was first discovered by Mobil Oil Research group, and it was named M41S [8]. The popularity of MSNs in the field of development of drug delivery agent is due to their easy synthesis process, high surface area and pore volume, easy single-step functionalization, tailorable particle size and morphology, good physicochemical stability and favorable biocompatibility [9]. The first successful application of MSNs as a drug carrier was conducted by Vallet-Regi and colleagues in 2001 encapsulating ibuprofen [10]. The FDA has recognized silica as “generally recognized as safe” (GRAS) for over 50 years, and it has been used in table pharmaceutical preparation as an excipient [11]. The most promising development is when small silica nanoparticles as imaging agents were approved by the US FDA for a human clinical trial [12]. This progress gives the scientists hope that MSNs as drug delivery agents can be applied in a real clinical situation.

In this review, we focused on the common methods in the fabrication of MSNs. Through our literature search, we found that most other review articles usually categorized the MSNs production based on the types, but for this review, we focused on the techniques. Under the fabrication of MSNs, we also covered its tunable properties and hybrid MSNs. This article also reviews the production of MSNs as smart drug delivery agents where passive and active targeting were covered. Besides that, in this section, stimulus-responsive MSNs were also reviewed, and we focused on the number of stimuli rather than the type of stimulus. In these recent years, through literature search, we discovered that MSNs have also been used as a multidrug carrier where single MSNs served as a carrier for two or more drugs.

## 2. Fabrication of MSNs

Mesoporous silica nanoparticles (MSNs) can be defined as solid materials that contain pores. The terms mesoporous in MSNs referred to the pore size. According to the International Union of Pure and Applied Chemistry (IUPAC), porous materials can be classified as microporous, mesoporous or macroporous. This is based on pore diameter where less than 2, between 2 and 50 nm and larger than 50 nm belong to a microporous, mesoporous and macroporous category, respectively. MSNs are very popular across various fields due to their properties such as tunable particle and pore size, high stability and rigid framework, large surface area and high pore volume, easy functionalization and simple fabrication [13].

To understand the attraction of MSNs, we should understand their history. The first mesoporous silica material, M41S, was discovered in the 1990s by a researcher in the Mobil Oil company. Like many breakthroughs, the discovery of mesoporous silica was accidental. M41S exhibited almost non-Brønsted acidity, thin amorphous walls, high surface area, large pore volumes and adjustable pore sizes [14,15]. In M41S, there are three main members, namely, MCM-41, MCM-48 and MCM-50. These can be differentiated through their pore geometry where MCM-41 shows hexagonal pore structure, MCM-48 has cubic mesostructured gyroidic phase *Ia3d*, and MCM-50 exhibits lamellar geometry that consists of silicate or porous aluminosilicate layers separated by surfactant layers [16]. Among these three, MCM-41 is the most widely studied as MCM-48 and MCM-50 are difficult to obtain and thermally unstable [15]. Since then, other types of MSNs have been discovered, for example, folded sheet mesoporous material-16 (FSM-16), Santa Barbara Amorphous family (SBA-n), Fudan University material (FDU), Korea Advanced Institute of Science and Technology (KIT), hollow MSNs and others [17]. In this section, we explore the current and new methods in synthesizing MSNs, including their functionalization.

### 2.1. Synthesis Techniques

**Figure 1:**
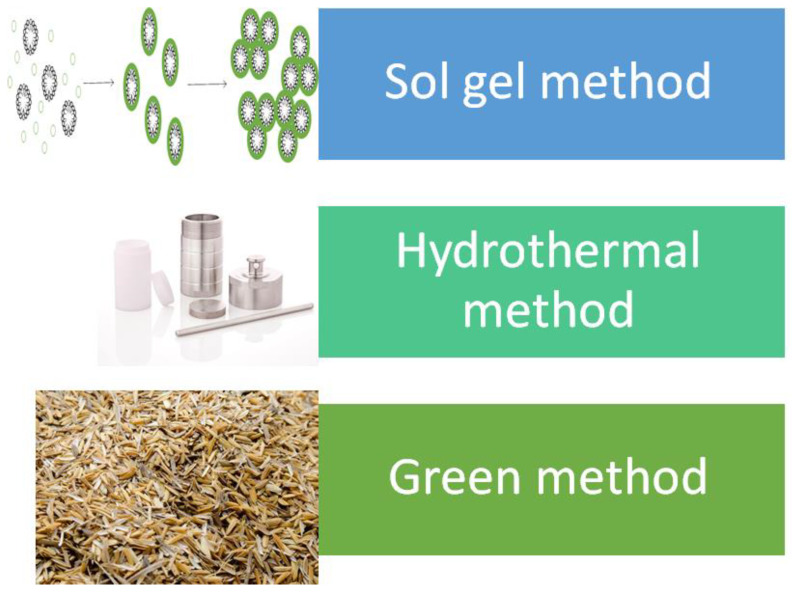
Common techniques in mesoporous silica nanoparticles (MSNs) synthesis.

In the synthesis of MSNs, there are three basic components: the template, typically a surfactant, which will serve as the agent to create the pores, the silica source to make up the walls surrounding the pores and acid or base to facilitate the formation. There are also additional compounds and/or conditions such as solvents, temperature, stirring rate, additives and others. These additional compounds and/or conditions will alter the properties of synthesized MSNs, and this will be further explained in the upcoming section. The synthesis techniques for the generation of MSNs can be classified into sol–gel, hydrothermal and a more recent green method. Table 1 and Figure 1 shows the summary of techniques in synthesizing MSNs.

#### 2.1.1. Sol–Gel Method

The formation of MSNs first began by Stöber with the formation of monodispersed spherical silica particles in the micron range. Stöber synthesis has four main components, which are water, base, alcohol and silica source. The dispersity of the particle was controlled through hydrolysis of alkyl silicates and subsequent condensation in the presence of alcohol [32]. To reduce the particle size to the nanometer range, various modifications were made. The formation of MSNs was first achieved by Grun et al., where they modified the Stöber method through the introduction of surfactant. Based on their results, the MSNs generated was in a spherical shape with MCM-41 properties [33]. The general process of synthesizing MSNs through the modified Stöber method consists of four steps as follows: Step 1: a mixture of water, template and base were mixed together. Step 2: then, the silica source was added to the mixture and stirred. In this step, the hydrolysis and condensation process takes place, and silica sol was formed. Step 3: the sol went through the aging process, and the gel was formed. Step 4: MSNs powder was obtained, and the template was removed through a calcination or acidic solvent extraction process [34]. Based on the process, the modified Stöber method in obtaining MSNs is considered as sol–gel method. Through this discovery, there are even more modifications made to achieve the desired MSNs.

Our literature search for the production of MSNs led us to notice that majority of articles focus on the sol–gel method for the synthesis of MSNs. Hwang and colleagues utilized polyethylene glycol (PEG) with a molecular weight of 3000 g/mol and sodium silicate as the template and silica source, respectively, in the generation of MSNs. The synthesis process was conducted under neutral pH with acetic acid as the pH adjuster. They were able to obtain MSNs with spherical morphology with a surface area value of 685 m^2^ g^−1^ [18]. In another work, a series of MSNs were obtained by using cetyltrimethylammonium chloride (CTAC) and tetrethylorthosilicate (TEOS) as the template and silica source. Triethanolamine (TEA) was used as the catalyst, and the pH of the solution is slightly basic. The reaction was relatively short (2 h), and the total volume of the solution was small, which makes the production collection process simple [22]. Zhou and coworkers successfully generated MSNs using cetyltrimethylammonium bromide (CTAB), silica fume and ethyl acetate as a template, silica source and catalyst, respectively. Their XRD analysis showed that the MSNs exhibited a peak at 2.2°, correspond to (100) plane reflection of hexagonal pore structure. Based on this result, they concluded that the pores were in ordered structure [35].

#### 2.1.2. Hydrothermal Method

The term hydrothermal was first used by a British Geologist, Sir Roderick Murchison, and he describes it as a process of minerals formation by hot water solutions rising from cooling magma. For chemists, hydrothermal synthesis is defined as a chemical reaction that takes place in a sealed container at elevated temperature and pressure [36]. Similar to the sol–gel process, the hydrothermal synthesis of MSNs proceeds through similar steps with slight alteration as follows: Step 1: water, template and catalyst were mixed together. Step 2: silica source was added. Step 3: in some cases, the mixture went through the aging process, while in the other, the mixture was stirred for a short period then transfer to a Teflon-lined autoclave. Step 4: The autoclave was heated at a certain temperature for a certain duration. Step 5: MSNs powder was obtained, and a template removal process was conducted. The advantages of MSNs obtained through the hydrothermal method are improved hydrothermal stability, mesoscopic regularity and extended pore size [37].

Relative few research articles in the last 5 years utilize the hydrothermal method in MSNs production. Song et al. reported the production of MSNs using CTAB and TEOS as a template and silica source, respectively. The synthesis process is similar to the Stöber method, where they used ammonia as the catalyst and ethanol to control the hydrolysis rate. The mixture was subjected to hydrothermal treatment with the conditions of 100 °C for 12 h. The synthesized MSNs exhibited a spherical morphology [24]. In another work, MSNs with fibrous dendritic or KCC-1 structure successfully obtained through hydrothermal synthesis. This work reported the synthesis process consists of a mixture of CTAB, TEOS, cyclohexane and urea. This mixture went through a hydrothermal process in a rotating oven at 120 °C for 4 h and 60 rpm. The MSNs exhibited spherical morphology with ordered fibers emanating from the center of the spheres [25]. MSNs with SBA-15 properties successfully produced with Pluronic F-127 and TEOS as a template and silica source, respectively, via hydrothermal method. The process was conducted in acidic conditions with hydrothermal conditions of 50 °C for 30 h. The MSNs obtained had a high surface area value of 1098 m^2^ g^−1^ [26]. Although this method is able to produce MSNs, it also poses several problems, such as it requires specific equipment and elevated temperature with long duration translated to high cost. Therefore, most research in recent years has opted for the sol–gel method.

#### 2.1.3. Green Method

Recently, a new method known as the green method has been proposed as an alternative method in the production of MSNs. The name green implied that the method is environmentally friendly and aims to employ waste products as the silica source. Abburi et al. managed to obtain MSNs by using hexafluorosilicic acid, waste from the fertilizer industry, as the silica source in the absence of a template. Based on the XRD results, the MSNs formed exhibit hexagonal and ordered mesoporous structure [27]. In another work, CTAB and ash of banana peels were used as a template and silica source, respectively [28]. Li and coworkers utilized modified amino acid as a template and TEOS as a silica source in the production of MSNs. The TEM images indicated that the MSNs contain pores with a “wormhole” arrangement. The MSNs obtained have a particle size ranging from 130 nm to 270 nm and surface area value ranging from 239–678 m^2^ g^−1^ [31]. It should be noted that although the method is considered green, the process may not obey the green chemistry principle. For example, the extraction of sodium silicate (silica source) from wastes such as rice husk, ash and others are complicated and conducted under extremely high or low pH.

### 2.2. Tunable Properties

MSNs have attracted much attention due to the possibility and ease of tailoring their properties. The three most common properties being manipulated are morphology or shape, particle size and pore size. For biomedical applications, the nanoparticle size must be within the range of 10 nm to 400 nm. Particles smaller than 10 nm will undergo renal clearance while larger than 400 nm cannot diffuse to the targeted cell to achieve a good activity. Previous studies have shown that the optimum particle size of MSNs for biomedical application is between 50–100 nm, as particles smaller than 50 nm resulted in non-specific distribution and optimal uptake exhibited by particles less than 100 nm [16]. There are various ways to control the particle size of the synthesized MSNs. Ortega and colleagues reported that they were able to control the particle size of spherical MSNs by varying the ratio between hydroalcoholic and aqueous medium where a higher portion of hydroalcoholic medium resulted in bigger particle size. They also studied the effect of pH and found that higher pH of reaction solution resulted in smaller particles [38]. In another work, the size of the spherical MSNs was controlled by changing the amount of triethanolamine (TEA), where reducing the amount of TEA used resulted in smaller particles [39]. A similar result was reported by the Möller research group [40]. However, our group and other work discovered a contradicting result where the particle size decreased with an increase of TEA [7,22]. Therefore, there may be other factors affecting the particle size. Yismaw and coworkers studied the effect of catalysts by comparing the MSNs produced using ammonia and TEA. MSNs produced using ammonia showed highly agglomerated spherical particles with a mean size of 420 nm. However, by changing the base to TEA, the particle size reduced to 186 nm, and they concluded that TEA served as both the catalyst and growth controlling additive [41]. Besides TEA and ammonia, sodium hydroxide is the most common reagent being used as catalysts in MSNs production. Many research articles have reported that the particle size of MSNs can be controlled by changing the amount of sodium hydroxide (varying the pH). Overall, these reports indicated that smaller particle was generated in high pH condition [42,43,44]. An alternative way to control the MSNs’ particle size is through its template. Previous work reported the production of MSNs by using various templates such as tannic acid, gallic acid, eudesmic acid, ethyl gallate and quercetin. They found that pores within the silica nanoparticles formed when tannic acid and gallic acid were used as the template. By changing the template from tannic acid to gallic acid, the particle size of synthesized MSNs increased from 240 nm to 600 nm [45]. Our research group also discovered that by increasing the alkyl chain length of the ionic liquid template, the particle size decreased [46]. The particle size of MSNs can also be controlled by utilizing grain growth suppressants. Poly(ethylene oxide)-poly(propylene oxide)-poly(ethylene oxide)(EO_20_PO_70_EO_20_) was added into the reaction solution as growth suppressant. By increasing its amount from 0 to 0.1 moles, the MSNs particle size decreased from 200 nm to 40 nm [47]. Synthesis temperature also affected the particle size of MSNs, where bigger particles were generated at elevated synthesis temperature [7,43]. Overall, the main components that affect the MSNs’ particle size can be summarized to the solution pH, template, presence of additives and reaction temperature.

Another property frequently tuned is the pore size or pore diameter of MSNs. Typical MSNs have a pore size of 2–3 nm, which is sufficient to serve as the carrier for small molecules [48]. However, it is not applicable to large molecules, which then limits the function of MSNs as drug delivery agents. Therefore, researchers have dedicated their work to expand the pore size. One study reported that the pore size could be adjusted by changing the concentration of TEOS in cyclohexane, and they managed to obtain MSNs with pore sizes ranging from 7.8 nm to 12.9 nm [49]. Liu and coworkers synthesized dendrimers like MSNs, where the MSNs exhibited center-radial pores with the gradual increase of pore size from the interior to the surface of MSNs. These MSNs was obtained through a biphasic stratification reaction system consist of chlorobenzene–water mixture with a pore size of 22 nm [50]. In another work, larger pore MSNs were obtained by varying the ethanol/water volume ratio. By decreasing the volume ratio, the pore size was increased up to 6.52 nm. The researchers proposed that this occurred due to the competitive behavior of ethanol and water. Water will promote the formation of a template’s micelles, and ethanol will weaken the assembly of the template; thus, by decreasing the volume ratio of ethanol/water, the enlarged pore can be obtained [51]. Interestingly, Ryu et al. reported a simple technique in fabricating MSNs with large pores where their method was based on rapid cooling of reactant then resuming the reaction. They anticipated that rapid cooling allowed complete separation between nucleation and growth process. They were able to obtained MSNs with a “wrinkled like” structure and pore sizes ranging from 15 nm to 33 nm [52]. Besides this, the most common way to increase the MSNs pore size is by employing a pore expander. MSNs with pore size up to 5.3 nm successfully obtained with trimethyl benzene (TMB) as the pore expanded [53,54]. Triisopropylbenzene (TIPB) is another common pore expander being used, and the MSNs obtained exhibit pore size up to ten and several nm [55,56]. Dendritic MSNs with extremely large pore size (up to 39.1 nm) was fabricated with sodium heptafluorobutyrate as a pore expander. The pore size increases proportionally with the amount of pore expander [57]. On the whole, the pore size can be tuned by controlling the ratio between solvent and water for the reaction medium, new synthesis condition and employment of pore expander.

**Figure 2:**
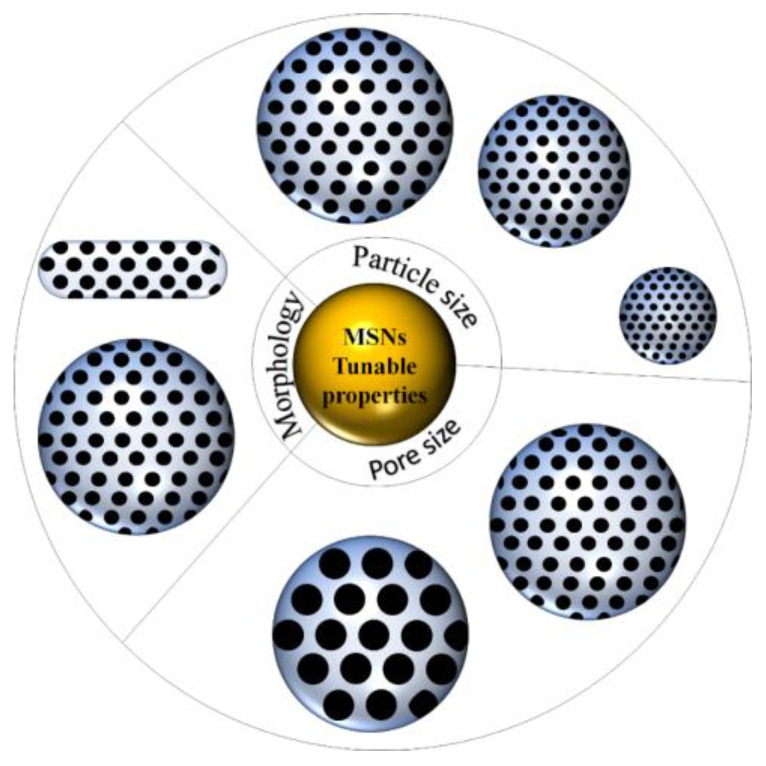
Schematic depiction of tunable MSNs’ properties.

Morphology or particle shape of MSNs has a great influence on the cellular uptake and biodistribution. This was studied by Huang et al., where they showed that particles with different aspect ratios greatly impact cellular function [58]. However, based on our search, MSNs with spherical morphology is the most commonly synthesized and studied. The change of MSNs morphology can be done by manipulating the synthesis parameters. MSNs with rod-like shape was successfully synthesized by varying the ratio between TEOS and 3-aminopropyltriethoxysilane (APTES). By increasing the ratio, the particle morphed from spherical to rod-like shape [59]. MSNs with nanorod shape successfully synthesized via hydrothermal technique under various acidic condition [60]. MSNs with spherical, ellipsoidal and rod-like shapes were synthesized in the presence of different ethanol amounts [61]. Song and colleagues synthesized a unique shape MSNs, yolk-shell, by using embedded organic phenolic resin as the template where, through the calcination, porous yolk-shell silica nanoparticles were formed [62]. The unique MSNs we discovered in our literature search were virus-like MSNs. These virus-like-MSNs was obtained through a single micelle epitaxial growth approach in a low concentration surfactant oil/water biphasic system. These MSNs consist of two parts, which are the interior spherical mesoporous silica cores and separated peripheral silica nanotube perpendicular to the core surface [63]. Our research group also managed to obtain MSNs with spherical, distorted spherical, raspberry and undefined shape by employing pyridinium ionic liquid with different anions as the template [64]. Overall, the tunable properties of MSNs can be summarized as in Figure 2. 

### 2.3. Hybrid MSNs

Hybrid MSNs is a relatively new term that can be defined as MSNs functionalized with inorganic or organic compounds [40]. The possibility of these hybrid MSNs is due to the surface chemistry of MSNs where silanol groups present on the silica surface. These silanol groups can be modified with various compounds, which can improve and expand the application of MSNs in the biomedical field [65,66]. In general, the functionalization of MSNs commonly proceeds through two methods, which are co-condensation (one-pot) and the post-synthetic process by grafting. In one-pot synthesis, the desired functional group is mixed with the surfactant and silica precursor during the nanoparticle’s formation, and this resulted in a homogenous amount of functional group attached. However, the MSNs obtained through this method cannot be calcined for a template removal as the functional group will be removed through the calcination process. For the grafting method, the functional group is introduced after the formation of MSNs, either before or after template removal. The advantage of this technique is the specificity of functionalization sites such as external surface, pore surface and pore entrance [67]. Based on our literature search, we discovered that the functionalization of MSNs could be classified according to the type and/or compound, such as functional groups, targeting agent, imaging agent, polymer or biopolymer, zwitterions and inorganic-MSNs hybrid. Besides functionalization, MSNs with an organic group within the silica framework has also been reported. There are two types which are mesoporous organosilica nanoparticles and periodic mesoporous organosilica.

#### 2.3.1. Functional Groups

Amine group is the functional group most commonly used to modify the MSNs surface. This group can serve as a precursor to graft other compounds [67,68]. Nguyen and coworkers utilized APTES via a post-synthetic grafting process to modify the surface of hollow MSNs (HMSNs) with amine groups. They also studied the effect of APTES concentration on the properties of HMSNs, and they found that the particle size increased with APTES concentration [69]. Our research group also did a project on modifying the MSNs surface via the post-synthetic grafting process. We reported that the surface of MSNs successfully grafted with amine, thiol or carboxyl functional groups [67]. Yismaw and colleagues modify MSNs with acrylate functional group, and they compared the effect of surface modification route (between co-condensation and post-synthetic grafting). The aim of their study was to graft the acrylate functional group on the outer surface of MSNs. Through a co-condensation method, they discovered that to achieve outer surface functionalization, the late addition of silane precursor resulted to selective surface functionalization. For post-synthetic grafting, the process was conducted prior to a template removal. They hypothesized with the presence of a template, the diffusion of silane precursor into the pore channel was restricted thus the silica oligomers graft on the outer surface instead [70]. Aldehyde functional group was successfully grafted on the surface of dendritic MSNs. The aldehyde group was grafted by using triethyoxysilyl butyraldehyde. This aldehyde group was grafted on MSNs surface to create a pH-responsive protein drug delivery system [71]. The disulfide group was successfully functionalized on MSNs via co-condensation method with bis(triethoxysilylpropyl)disulfide (BTES) as the functional group precursor [72]. Interestingly, one research group reported on the functionalization of MSNs with two compounds via the grafting process. This group grafted diethylenetriamine group and tin compound on the MSNs in a successive step. Their result indicated that the functionalization occurred inside the pores of MSNs [73].

#### 2.3.2. Targeting Agent

In order to improve the MSNs properties as a drug delivery agent, targeting agents can be functionalized onto its surface. Through this, the therapeutic efficiency is increased as the targeting agent can differentiate between disease and normal cells [74]. Liu and coworkers reported the usage of sulfated glycosaminoglycan (ChS) as a targeting agent, and it was grafted on the surface of MSNs through several steps. The attachment of the targeting agent began with the functionalization of the amine group, which was then oxidized to a carboxylic group. This carboxylic group was used to attach cystamine to the MSNs surface, and cystamine was further reacted to the precursor of targeting agent to functionalize MSNs with ChS [75]. In another study, the targeting ligand arginine-glycine-aspartic (RGD) peptide was grafted on MSNs surface. Similar to the previous work, to graft the RGD peptide, MSNs were first functionalized with amine groups, then converted to a carboxyl group. This carboxyl group will react with the amine group of RGD peptide to covalently attach the targeting ligand on the MSNs surface [76]. Hyaluronic acid (HA) is also a targeting agent that is recognized by the CD44 receptor on the tumor cells. One work reported the grafting of HA with various molecular weights on MSNs, and the effects of molecular weight on the carrier internalization were studied [77]. Chen and colleagues reported on the attachment of targeting agent, lectin Ulex europaeus Agglutinin-1 (UEA1), to specifically target human colorectal adenocarcinomas, adenomas and polyposis coli. Interestingly, this targeting agent does not recognize normal human colorectal cells [78].

#### 2.3.3. Imaging Agent

MSNs functionalized with an imaging agent is beneficial to visualize the carrier movement throughout the system and their internalization in the cells. The most common imaging agent is fluorescein isothiocyanate (FITC). The co-condensation method was used to attached FITC to the MSNs [68]. FITC was used as an imaging agent due to its intrinsic brightness, and with its functionalization on MSNs, the proximity-induced self-quenching can be minimized, thus resulted in a brighter contrast agent [78]. In another work, natural product emodin (EO) was used as a fluorescence agent concurrent with another two fluorescence agents, N-methyl isatoic anhydride and lissamine rhodamine B sulfonyl moieties. By combining different imaging agents, quenching interaction can be prevented, and visualization with different wavelengths was achieved [79]. Besides fluorescence agent, T1 MRI contrast agent has been gaining more attention as an imaging agent. Du and colleagues reported the hybridization of MSNs with manganese oxide nanoparticles as a T1 MRI contrast agent. They selected manganese oxide nanoparticles as imaging agents as it showed less toxicity due to the properties of manganese as a physiological regulator of enzymes [80].

#### 2.3.4. Polymer

For the purpose of drug carrier, MSNs are frequently functionalized with polymer as it has a higher probability to improve the overall performance and therapeutic effect of the carrier [81]. The class of polymer to be functionalized on MSNs will be based on the desired outcome such as biocompatibility, specific targeting, stimuli-responsive or sustained release of drugs from the MSNs [82]. However, in this section, we will be focusing on the polymer functionalized MSNs, while the purpose of polymer functionalization will be further explained in the upcoming sections. Plohl et al. successfully functionalized MSNs with branched polyethyleneimine (bPEI) via post grafting method. The particle size of functionalized MSNs was between 80 to 180 nm, with surface area ranging from 706 to 724 m^2^ g^−1^ [83]. Polyethylene glycol (PEG) is one of the most commonly used polymers to functionalized MSNs. With PEG functionalization, the carrier can prevent non-specific protein adsorption, increases the stability and blood circulation time, which are important for an effective drug delivery agent [84,85]. In a study, PEG was functionalized on MSNs surface through previously modified MSNs, which contain both amine and disulfide groups. They proposed that PEG was covalently bonded to the MSNs via disulfide bond [85]. Besides synthetic polymers, as stated previously, the biopolymer can also be used to functionalized MSNs. Chitosan was functionalized onto the MSNs surface via grafting method to crease a pH-responsive system. The particle size for this chitosan functionalized MSNs ranging between 80 and 130 nm [86].

#### 2.3.5. Zwitterion

MSNs can also be functionalized with zwitterions, and through this, the materials are considered as bioinert and biocompatible Zwitterion can be defined as an electrically neutral compound where the positive charge and negative charge are balanced to each other. They are hydrophilic and MSNs coated with them exhibit a similar structure to a cell phospholipid membrane. The different charges on the surface facilitate the interaction of MSNs with the cell membrane, which can increase the internalization of the MSNs into the cell [84,87]. Research on functionalizing MSNs with zwitterion is relatively new. Thus, the number of articles on it is low. In one study, zwitterion functionalized MSNs was synthesized by modifying the MSN surface with deoxycholic acid (DC) and sulfobetaine 12 (SB12). First, the MSNs were functionalized with amine groups. These amine groups react with DR to create a hydrophobic surface. Then SB12 was self-assembled on the hydrophobic surface to create a neutral and hydrophilic coating. The functionalization was done through the post-synthetic grafting method [87]. In another study, amphiphilic monomer 2-methyacryloyloxyethyl phosphorylcholine (MPC) was polymerized via reversible addition–fragmentation chain-transfer (RAFT) on the MSNs surface to create zwitterion functionalized MSNs [88]. Wan and coworkers successfully synthesized zwitterion functionalized MSNs using photopolymerization of 2-[dimehyl-[-(2-methylprop-2-enoyloxy)ethyl]azaniumly]propane-1-sulfonate polymer (pSBMA) via grafting method. The particle size of the functionalized MSNs was relatively small, which was approximately 100 nm [89].

#### 2.3.6. Inorganic-MSNs

Besides organic groups mentioned in the previous section, MSNs have also been functionalized with inorganic groups such as metal and metal oxides. Several reports have shown a successful combination of gold and MSNs. Gold nanoparticles have been used as a cap to close the pores of MSNs, and this was to create a stimulus active release carrier [90]. Core-shell structures consist of a gold core, and MSN shells have been reported. The gold core was coated with MSNs shell via a sol–gel method of MSNs production. Interestingly, there were two morphologies reported for gold-MSNs core–shell structure, which are spherical and rod-like shape [91,92]. Marcelo and colleagues reported on the production of Janus nanoparticles consist of gold and MSNs. The term Janus nanoparticles refer to the side-by-side position of gold and MSNs [93]. Another common inorganic-MSNs hybrid is iron oxide and MSNs hybrid. Iron oxide nanoparticles exhibit good magnetic properties, and they were found to be frequently hybridized with MSNs. Due to their magnetic properties, the iron oxide-MSNs hybrid can be controlled with magnetic fields. This is important in the creation of a drug delivery agent where carriers consist of iron oxide-MSNs hybrid can be moved to the target location through a magnetic field [94,95,96,97]. Both titanium and titanium dioxide have been reported to be functionalized with MSNs [98,99,100]. The titanium dioxide was grafted on the MSNs by mixing titanium dioxide with MSNs and stirred for 10 h. Then the product was collected and calcined [98]. In another work, two types of titanium dioxide-MSNs hybrid were prepared where one with a hollow structure was produced. The titanium dioxide was grafted on MSNs using tetrabutyl orthotitanate. The create hollow titanium dioxide-MSNs hybrid, an additional step of etching was added [100]. There were also report on MSNs hybridized with silver, magnesium, calcium, gallium, copper, manganese and molybdenum disulfide [101,102,103,104,105,106].

#### 2.3.7. Mesoporous Organosilica Nanoparticles (MONs)

Other than surface functionalization stated in the previous section, a new class of organic–inorganic hybrid MSNs was discovered independently by Ozin’s, Stein’s and Inagaki’s groups circa 1999. This new class of hybrid MSNs showed that the organic components are present within the silica framework instead of the MSNs surface [107]. This material can be classified into two, which are mesoporous organosilica nanoparticles (MONs) and periodic mesoporous organosilica (PMO). The production of MONs and PMOs are similar to the sol–gel synthesis of MSNs, with the only difference being the silica source used. For MONs, the combination of tetraalkyoxysilane and organosilane is used, while pure organosilane is used as a silica source to generate PMOs. The type of organosilane used in MONs and PMOs production is typically bridged organosilane, where the organic group is placed between two or more silicon atoms. The structure of bridge organosilane is R[Si(OR′)_3_]*_n_* where *n* ≥ 2. When n is equal to two, the organosilane is known as bis-silane, and when n is greater than two, it is known as multi-silane. The R and OR’ are referring to the functional linkers and hydrolyzable groups (typically ethoxy or methoxy groups) [107,108,109]. Through the aging process, this organosilane with or without silane group will undergo a condensation process around the template, and a silica framework is formed. The materials then undergo a template removal process. It should be noted that to preserve and protect the organic group in the framework, a mild removal process, such as solvent extraction or dialysis approach, should be utilized instead of the calcination process, which is commonly used for template removal in MSNs [108,109]. It is relatively rare to find reports on the successful production of MONs and PMOs with monodisperse and uniform nanoparticles. This is due to the properties of organosilane such as size, wettability, electric charge and chemical characters, which can affect its interaction with the template, thus affected the properties of generated MONs and PMOs. Therefore, the selection of organosilane is crucial to produce MONs and/or PMOs with desired characteristics for specific applications [107,108].

In the production of MONs, thioether is the most common organic group added within the silica framework. The silica source used to add this thioether group is bis[3-(triethoxysilyl)propyl]tetrasulfide (BTES). With CTAB as the template, a silica source consists of a mixture of TEOS and BTES was added to the mixture under the basic condition to form targeted MONs. Two of the recent reports showed that the MONs produced exhibited spherical morphology [110,111,112]. Interestingly, rather than using CTAB as a template, He and coworkers utilized chlorohexidine both as a template and cargo of the synthesized MONs. Instead of proceeding to the template removal process, they utilized the synthesized MONs with the template as a completed drug delivery agent [111]. Besides that, hollow MONs has also been synthesized with BTES as the silica source. The hollow MONs with thioether bridge began a surfactant-assembly sol–gel process with CTAB as a template and TEOS and BTES as a silica source. Through hydrothermal treatment, multiple small cavities were formed due to selective etching. Then, the template was removed using an acidic ethanol extraction process, and the MONs form exhibited a hollow structure with multiple cavities [113]. In other work related to the production of hollow MONs with thioether bridge reported its formation through the production of a silica nanoparticle (SiO_2_ NPs). First, SiO_2_ NPs were produced through the Stöber method and then the core-shell structure was formed with a thioether group within the framework. The shell was formed through the typical synthesis of sol–gel synthesis of MSNs except with BTES as an additional silica source. To form a hollow core structure, the SiO_2_ NPs were etched using hydrofluoric acid (HF). The TEM results showed that the hollow MONs form exhibited single cavities with a porous shell structure [114]. Li and colleagues successfully produced MONs containing thioether group with nanorod morphology through varying the amount of water in the reaction [115]. Interestingly, nanosheet MONs with thioether was synthesized recently. They reported that to produced nanosheet morphology, first SiO_2_ NPs were produced, and it underwent a high-intensity ultrasound to break them into small fragments (sSiO_2_ NPs). These sSiO_2_ NPs were then sandwich with a mesoporous organosilica layer. Then MONs nanosheets were then generated through selective etching of sSiO_2_ NPs using sodium borohydride [116]. Ethane organic moiety has been reported to be incorporated in the silica framework of MONs by using 1,2-bis(triethoxysilyl)ethane (BTEE) as one of the silica sources [112]. Rather than typical spherical MONs, Du and a coworker successfully produced MONs containing ethane group with nanobowl morphology. They proposed that the nanobowl morphology formed due to the uniform and dynamic coating of BTEE on MSNs surface, simultaneously gradual dissolution of MSNs core, regrowth and reassembly of a small portion of dissolved species which resulted in nanobowl morphology [117]. Tao et al. reported the production of MONs containing ethane group with yolk-shell structure. To form this structure, an etching process using sodium carbonate was conducted, which transforms the MONs with a dark core encapsulated with a gray hollow shell [118]. A combination of two organosilanes to produce hollow MONs has been reported. In this work, the MONs was characterized with the core contained double hollow shells, thioether and ethane group were separately incorporated framework. MONs were produced via the hydrothermal method, and after the treatment, two-layered mesostructured organosilica nanospheres were obtained with thioether in the interior and ethane on the exterior layer [119]. Benzene group in a silica framework of MONs also has been reported. The study showed that the MONs produced exhibited high hydrophobicity [120]. One of the interesting MONs was produced with ferrocene in the framework. To produce this, the ferrocene was first modified to attach the silyl group. Then, this modified ferrocene was used as a silica source together with TEOS to produce the desired MONs. The amount of modified ferrocene was varied, and it was discovered that with increasing ferrocene amount, the morphology change to macroscopic. The reason for this was due to the structure of ferrocene which is large and can affect the hydrolysis process [121].

As stated previously, another class of organic–inorganic hybrid MSNs is PMOs. For PMOs production, the silica source consists of the only organosilane. There were not as many reports on PMOs compared to MONs. This is due to the challenging aspect of PMO production in terms of porosity. It was discovered that the high content of organic group hinders pores formation [109]. There were several studies that reported on the fruitful formation of PMOs by using pure organosilane. Laird and coworker managed to synthesize PMOs containing benzene organic moiety using 1,4-bis(triethoxysilyl)benzene (BTEB) as the silica source, Pluronic P123 as the template and the reaction was conducted in acidic condition. Through variation of reactants’ addition sequence, monomodal and bimodal porosity were generated [122]. In other work, hollow PMOs were successfully synthesized using BTEB and BTEE separately. They utilized pentablock copolymers as a template, and the production of hollow PMOs was conducted in acidic conditions. Both PMOs containing benzene and ethane organic groups, respectively, exhibited spherical morphology with particle size approximately 25 nm [123]. PMOs with two organic moieties successfully reacted with benzene and pyridine organic groups present in the silica framework. The silica sources to form the PMOs were a combination of 1,4-bis[(triethoxysilyl)vinyl] benzene and 2,5-bis[2-(triethoxysilyl)vinyl] pyridine. Through the variation of a silica sources ratio, the particle morphology changed from spherical to a rod with the increase of pyridine silica source [124]. Amine is another organic moiety successfully integrated into the silica framework to form PMOs. The silica source to form amine-containing PMOs is bis-[(3-triethoxysilyl)propyl] amine (BTESPA). By using 100% BTESPA as the silica source, monodispersed spherical nanoparticles with a diameter of 500 nm were generated. It was also reported that the amine groups were expected to be evenly distributed throughout the PMOs skeleton [125]. Dual organic group-containing PMOs were also produce using (3-trimethyoxysilylpropyl)-diethylenetriamine and BTEE as the silica sources. The number of silica sources was varied [126]. Interestingly, PMOs containing porphyrin has been reported to be successfully produced. Prior to the synthesis of PMOs, the porphyrin was first modified with octatriethoxysilyl, and this modified porphyrin was used as a silica source to form PMOs with porphyrin in the backbone [127]. In summary, most research related to mesoporous organosilica focused on the formation of MONs rather than PMOs. This may be due to the ease of obtaining MONs with desired properties compared to PMOs, which relatively harder to tailor to desire.

## 3. MSNs as Smart Drug Delivery Agent

The properties of MSNs such as high surface area, biocompatibility, easy functionalization and others make them favorable to be applied as a drug delivery agent. The high surface area allowed a high concentration of drugs to be incorporated, but premature release prior to reaching the target is a definite concern. However, due to the presence of pores in the MSNs, the drug can be loaded into the pores, and the pore opening can be closed with other molecules. To make the MSNs more effective carrier, the gate of the pore can be opened through a specific stimulus. Besides that, these MSNs can also be a multifunctional carrier, and this will be explained in the latter section. In this section, we will discuss and focus on studies that have reported on the production of MSNs as drug delivery agents and their evolution from simple passive targeting of drug delivery to within stimulus-responsive drug delivery methods.

### 3.1. Passive Targeting

The term passive targeting can be defined as the accumulation of nanoparticles in solid tumors. The foundation of passive targeting was discovered by Matsumura and Meda around 1986. They found two key observations to passive targeting: (1) spontaneous accumulation of drug carrier in areas of solid tumors with leaky vascular and (2) retention of the carrier due to compromise lymphatic drainage. With these two observations, the concept of enhancing permeability and retention (EPR) effect was formed [128]. To further understand the basis of the EPR effect, we must first understand the pathophysiology of the tumor. Solid tumors grow at a rapid rate, and this comes with high nutrients and oxygen demand. Thus, new blood vessels and neovasculature were formed, and this is term as angiogenesis. These new blood vessels often exhibit disorganized course, irregular, discontinuous epithelium and structurally different from healthy vessels. Due to this, the nanoparticles can leak between the gaps and enter the tumor. This stage is referring to the enhanced permeability of the EPR effect. It should be noted that solid tumor has poor lymphatic drainage. Molecules smaller than 4 nm can diffuse back to the bloodstream, but the nanoparticles are impeded due to their larger particle size, thus retain in the solid tumor. This part refers to the retention of the EPR effect [128,129].

For MSNs to be used as a drug delivery agent through passive targeting, there are several parameters that must be controlled, which are the particle size, particle shape and surface properties. The particle size must be larger than the renal clearance level but small enough to be able to diffuse to the tumor cell through the leaky vessel, which is approximately between 50 nm to 300 nm. A previous study showed the non-spherical nanoparticles could reduce phagocytosis, which leads to longer circulation. However, it is difficult to conclude that particle size is the main variable as there are many techniques and materials used to produce MSNs. Therefore, particle size may not be the main parameters that need to be controlled to achieve a good EPR effect [129]. Surface properties of MSNs are the most important aspect for a good EPR effect. With surface modification, the MSNs can avoid the reticuloendothelial system (RES), thus preventing clearance. Polyethylene glycol (PEG) was found to be commonly functionalized with MSNs as it can reduce RES uptake and improve the overall stability of MSNs, which can help to enhance the EPR effect [130,131]. Although this passive targeting through the EPR effect is a good way to deliver drugs, it still poses various limitations. The mechanism of nanoparticle entrance to the tumor is more complex, and this EPR effect is pronounced in small animal xenograft tumor models, which are used in the in vivo study. Although this EPR effect occurs in humans, it varies greatly between a person and tumor type [132]. Even for the same tumor, the vascular permeability and lymphatic drainage differ for each area. This heterogeneity will limit the even distribution of carriers, thus lower the therapeutic effect [128]. Therefore, a better drug delivery agent is needed to overcome this issue by passive targeting.

### 3.2. Active Targeting

**Figure 3:**
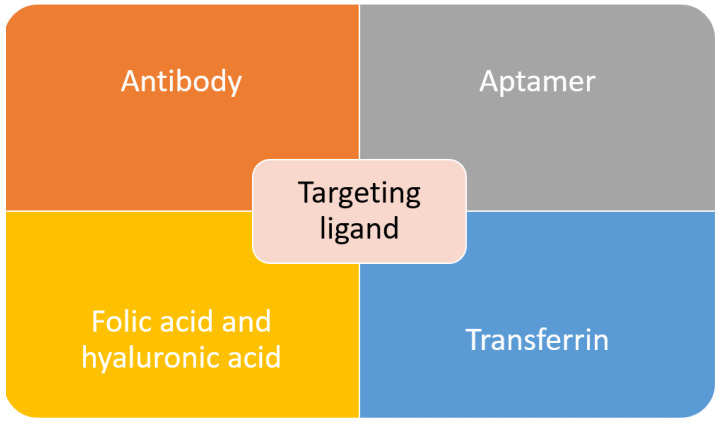
Common targeting ligands functionalized on MSNs.

Active targeting is employed to overcome some of the problems posed by passive targeting. This is achieved through exploiting the tumor cell-specific receptors. On tumor cells, a receptor is highly expressed. Thus, it is a sensible target to achieve active targeting. By decorating or functionalizing the MSNs surface with a targeting ligand which can interact with the receptors, the uptake and EPR effect can be enhanced as more carrier accumulate at the tumor site which will increase the efficacy of the treatment [133,134]. Besides that, by introducing the targeting agent, drug delivery is not completely dependent on the EPR effect, and this active targeting can reach hematological malignancies, small metastatic tumors and others that do not exhibit the EPR effect [135]. In order to achieve a good targeting strategy, the ligand density is the key factor. In the case of high-density of targeting ligand, it will cause other effects such as increased the clearance possibility, increased of particles size which will minimize the EPR effect, a steric hindrance which will reduce the binding capability and reduced cellular uptake [136]. The common targeting ligands functionalized on MSNs are summarized in Figure 3.

Antibodies, also known as an immunoglobulin (Ig), are one of the important factors to develop an active targeting drug delivery agent. Through the identification of an antigen specifically overexpressed on cancer cells, complement antibodies can be used to lead the carrier to the tumor site. Antibody targeting agent posed a few advantages over other compounds due to its specificity and selectivity. It typically exhibited “Y” shape protein, which consists of two heavy and two light chains. The two arms served as recognition sites for antigen, while the stem serves as effector functions. There are several factors that will affect the efficacy of a targeted system, such as antibodies’ configuration, origin and mode of linkage to the carrier [136,137,138]. There have been many reports on the functionalization of the antibody as a targeting agent to the MSNs. One work report on targeting prostate cancer through MSNs conjugation with prostate-specific membrane antigen (PSA) antibody. The PSA antibody is attached to the MSNs through the esterification reaction between the carboxyl group and an amino group [80]. In another work, to target retinablastoma (RB), an antibody was conjugated with MSNs to deliver carboplatin. It was found that in RB, there is overexpression of epithelial cell adhesion molecule (EpCAM), and its inhibition will result in poor cell proliferation. Therefore, this work reported on the conjugation of EpCAM antibody on MSNs through esterification with the carboxyl group on MSNs surface [138]. To target HeLa cells, a monoclonal anti-human epidermal growth factor receptor (EGFR) antibody, clone AT6E3 was conjugated to MSNs composite. The antibody was attached to the graphene oxide (GO) surface of MSNs composite. The result showed that with antibody conjugation, the carrier was internalized and retained in HeLa cells, and this indicated the effectiveness of the antibody in cancel targeting and treatment [139]. The FDA-approved antibody trastuzumab, which targets the HER2 receptor, was conjugated with MSNs to target breast cancer cells [140,141,142]. Although antibodies are effective in targeting the desired cancer cell, their high cost and potential adverse immune response have caused the research of another alternative targeting agent.

Another class of compound that can be used as a targeting agent are aptamers. They are defined as short RNA or single-stranded DNA oligonucleotides or oligopeptides (5–80 amino acids), which can bind to specific targets through folding into unique three-dimensional conformations [143,144]. Aptamers have unique characteristics such as small size, high stability, structure flexibility, ease of synthesis, and low or no immunogenicity, and these make them a great targeting agent [145]. In recent work, MSNs were successfully functionalized with AS1411 DNA aptamer as a targeting agent towards colorectal adenocarcinoma. This aptamer was bonded to the PEG that covering the MSNs surface [146]. DNA aptamer specifically targeting mucin-1 (MUC-1), was successfully functionalized to the MSNs. MUC-1 is highly expressed on the surface of breast cancer, ovarian and lung cancer and in this study, the efficacy of the carrier was determined against MCF-7 human breast cancer cell [147]. Tuna and coworkers reported on the usage of aptamer as both the capping and targeting agent of MSNs carrier. The aptamer, nucleolin AS1411, was used to close the MSNs pores to entrap the cargo, carbendazim. This carrier was tested against human cervical adenocarcinoma cells. The results showed that MSNs with aptamer gate exhibited a 3.3-fold increase of toxicity to the target cells compare to pure carbendazim [148].

Through our literature search, we discovered that the most commonly used targeting agent is folic acid. It is also commonly known as vitamin B9, and it can bind with folate receptors. There are three isoforms of folate receptors, namely hFRα, hFRβ and hFRγ. hFRα is the most commonly targeted as it is overexpressed across various cancers such as uterus, ovary, breast, cervix, kidney, colon and testicular. Various works have reported on the functionalization of folic acid on MSNs surface to target breast cancer [149,150,151], lung cancer [152,153], leukemia [154] and pancreatic cancer [155]. Although folic acid is an effective targeting agent, the presence of folate receptors on normal cells can interfere with the efficacy of the drug delivery agent. Another receptor that is overexpressed in solid tumors such as pancreas, breast and lung cancer is a cluster of differentiation-44 (CD-44). The commonly used targeting agent for this receptor is hyaluronic acid. It is an anionic nonsulfated glycosaminoglycan component of the extracellular matrix. It is also biocompatible and nonimmunogenic. Thus, it is frequently used in biomedical applications. There have been several works that report on the functionalization of hyaluronic acid on MSNs as a targeting agent [156,157,158,159]. Interestingly, one study reported on the use of hyaluronic acid as both pore closing of MSNs and targeting agent of the carrier. To close the MSNs’ pores, a pH-sensitive group was bonded between the MSNs and hyaluronic acid. The carrier will reach the target site using hyaluronic acid as the ligand, and at the tumor site where the pH is lower, the bond connecting hyaluronic acid with MSNs will be broken, and the drug will be released [160].

Transferrin is another type of ligand that can be used to target cancer cells with overexpression of transferrin receptors. Transferrin can facilitate the nanoparticle entrance to the cancer cells through receptor-mediated endocytosis pathway [137]. Pallares and colleagues reported on the delivery of MSNs carrier using transferrin as targeting ligand towards breast cancer [161]. A derivative of transferrin, ferritin, also has been used as the targeting agent to target the transferrin receptor. In this work, ferritin was used as both the gate for the MSNs pores and targeting agent [162]. Pancreatic cancer cell was successfully targeted using transferrin conjugated polymer-coated MSNs with gemcitabine as the anticancer drug [163]. There were many other works that utilized transferrin as a ligand, but it will not be covered in this review. The final popular targeting ligand is arginine-glycine-aspartic acid (RGD) peptide, which can target the integrin receptor. Integrin receptors are found to be overexpressed on angiogenetic endothelial cells and certain tumors, but they are absent in normal cells. This makes them an attractive receptor to be targeted in cancer therapy using RGD peptide [133]. Recently, Yan and coworkers reported on dual ligand using RGD peptide and folic acid functionalized MSNs to target human breast cancer MCF-7 cell [164]. Similar to previous reports, peptide-containing RGD motif was used as both the gate to cover the MSNs pores and targeting agent [165]. Overall, there are various targeting agents that can be used to improve the properties of MSNs as drug delivery agents. Although active targeting is able to improve the efficacy of anticancer drug delivery, the risk of premature release is still possible. Therefore, the research to discovering an improved carrier has evolved towards a stimulus-responsive system, which will be further discussed in the upcoming section.

### 3.3. Stimulus Responsive

**Figure 4:**
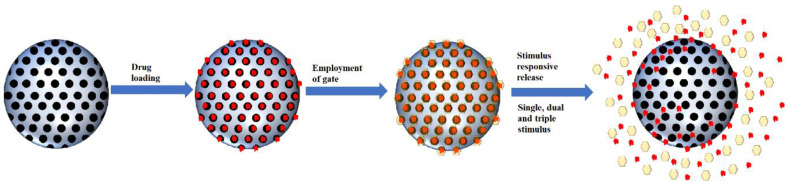
Schematic illustration of smart MSNs with the stimulus-responsive system.

The development of MSNs as drug delivery agents evolved from passive targeting, active targeting and now stimulus responsive system. Based on our literature search, the general form of a stimulus-responsive system utilizes both passive targeting and active targeting simultaneously with an additional gate to trap the cargo. The weakness of both passive and active targeting is premature to release of cargo during circulation; therefore, a gate was employed to close the pores of MSNs. This gate will be open once it reaches the target sites through a specific stimulus (Figure 4). The type of stimulus can be classified into two categories: internal and external stimulus. However, in this review, we will be categorizing the responsive stimulus system into the number of stimuli, which are single stimuli and multiple stimuli.

#### 3.3.1. Single Stimulus

As was mention previously, there are two types of stimuli, which are internal, such as pH, redox and enzyme, and external stimuli, such as light and temperature. In this section, we will be covering the common stimulus for both types of stimuli with the focus on the single-stimulus-responsive-MSNs system. Table 2 summarizes the research that utilized single-stimulus-responsive-MSNs for delivery of the anticancer drug. The gatekeeper and stimuli type are also indicated.

pH-responsive MSNs is one of the promising vehicles to deliver anticancer drugs to the targeted region. This system was developed through the exploitation of pH values in most tumors, which is lower compared to normal cells. The lower pH (around 6.5) value is due to the Warburg effect, where the cancer cells produce energy through glycolysis with or without oxygen, and this leads to the production of acidic lactate [197]. Furthermore, after the internalization of the carrier to the cancer cells, it will be exposed to even more acidic media in endosomal (pH 5.5–6.0) and lysosomal (pH 4.5–5.0). Kundu and colleagues reported on the delivery of umbelliferone, a plant-derived natural product, using pH-sensitive MSNs. The pores of MSNs were capped with polyacrylic acid, pH-sensitive polymer, and the surface was grafted with folic acid as the targeting agent. The drug was released under acidic pH due to the less electrostatic interaction and dissociation of the amide bond between the MSNs and the polymer [150]. In another work, poly-l-lysine gated MSNs were used to deliver doxorubicin. In a neutral environment, poly-l-lysine shrunk, which formed a dense barrier on the pore entrance. As the pH reduced, the polymer is swollen, and the drug was released [166]. One work reported on the usage of zinc oxide quantum dots as the gate to trap doxorubicin in the MSNs pores. The drug released at lower pH as the zinc oxide quantum dots dissolve in acidic condition and this lead to drug released [169]. Chen and coworkers successfully produced pH-responsive MSNs with “release-stop-release” properties. Typical pH-responsive MSNs will be activated in acidic conditions, and the process is not reversible. In this work, they managed to produce pH-responsive MSNs with reversible function. They utilized poly(tannic acid) as the gatekeeper with tetrethylenepentamine (TEPA) as a crosslinker. In acidic conditions, TEPA was protonated, thus increasing its hydrophilicity. This resulted in poly(tannic acid) swelling and substantial drug release. As the pH increased, the TEPA deprotonated and poly(tannic acid) will become dense again, thus closed the pores of MSNs. The authors stated that through this system, the side effect of the leftover drug could be minimized as safely excreted out [167].

Utilizing redox potential is another pathway to develop a stimulus responsive drug delivery agent. In a cancerous cell, the production of reactive oxygen species (ROS) is elevated due to genetic mutation, mitochondrial dysfunction and different metabolism. To overcome this, the production of ROS scavenger, the prominent being tripeptide glutathione (GSH), is also increased. It should be noted that GSH also presents in normal cells, but in cancerous cells, it contributes to the cancer progression and responsible for the resistance increment of radio- and chemoresistance of cancer cells [136]. It was found that the concentration of GSH is significantly higher in cancer cells (approximately 2–20 mM) compared to the extracellular part (2–20 nM) [198]. By utilizing the difference in the GSH concentration, the release of drugs from redox responsive MSNs upon cancer cell entrance will occur. One work reported on the utilization of redox potential to produce a redox responsive system with chitosan as the gate to trap the drug in the MSNs pore. Interestingly, this work used a fluorescent agent as the anchoring molecule which binds the chitosan with MSNs. This fluorescent agent was reported to be sensitive to GSH. Once the carrier entered the cancer cell, this fluorescence agent will be cleaved, and chitosan will be released from the carrier. Therefore, the drug will be released to treat the cancerous cell [172]. Thiol-linker is one of the most utilize linker to develop redox responsive MSNs. This linker will be broken by GSH, which will lead to the drug release. Zhang and coworkers reported on the redox responsive system with gold (Au) nanoparticles as the gate to the MSNs. The Au was bonded to the MSNs surface using a thiol-linker and exposure of this carrier to high GSH concentration’s environment, the linker was broken, and the drug, doxorubicin, was released [90]. In another work, the redox responsive MSNs was developed through the layer-by-layer assembly of curcubit-6-uril and cyclopentyl methylamine polymer, which served as the gate to cover the MSNs pores. This work studied the effect of layers’ numbers on the drug release activity with redox stimulus. In the presence of GSH, the percentage of drug released from the carrier decreased with increased layer number. They concluded that the cargo release rate could be manipulated through the variation of polymer layers [173].

Another stimulus-responsive system that is popular is the enzyme responsive MSNs carrier. Utilization of enzyme as stimulus poses several advantages such as high chemical selectivity, substrate specificity and mild condition. For cancer treatment, there is a specific enzyme present in this tumor microenvironment. Therefore, by employing a carrier with a component that sensitive to the present enzyme, a control released system through enzymatic cleavage is obtained [199]. Cai et al. reported on the production of enzyme responsive MSNs with chitosan as the gate responsible for trapping the drug in the MSNs pores. The component that was sensitive to the enzyme is the azo bond linked to the MSNs and chitosan. The results showed that the azo bond was broken in the presence of colon enzyme secreted by colonic microflora [179]. In another work, the same enzyme was used as a response to MSNs with guar gum as the pores’ cap. The colon enzyme degraded the guar gum, and the drug, 5-fluorouracil, was released [181]. Functionalization of MSNs with hyaluronic acid was found to be an enzyme responsive carrier in addition to active targeting [183]. Hyaluronic acid as a targeting agent can bind with CD-44 receptor, which is found in most cancer cells, and degraded by the enzyme hyaluronidase. One work used hyaluronic acid as both the gate and targeting agent [182]. Zhou and colleagues reported on the production of MSNs conjugated with hyaluronic acid and collagen I matrix for enzyme responsive system. Interestingly, this carrier was reported to be responsive to two enzymes, hyaluronidase and metalloproteinase-2. Through drug released studies, it was found that in the absence of enzymes, no drug was detected, while in the presence of an enzyme, up to 75% of the drug was released [184].

Other than internal stimulus, as stated previously, an external stimulus such as light and temperature were found to be commonly applied in the development of a responsive delivery system using MSNs. To generate light responsive MSNs, functionalization using a photoactive group is a need. The trigger for drug release will be light with various wavelengths such as ultraviolet (UV), visible (Vis) and near-infrared (NIR) [133]. Wang and coworkers reported on UV responsive MSNs carrier for anticancer treatment. The pore of MSNs was capped with α-cyclodextrin with hydrazone bond linking the azobenzene functionalized MSNs and α-cyclodextrin. Through UV irradiation, the azobenzene conformation changed, which led to the dissociation of α-cyclodextrin and the drug, doxorubicin, released [188]. Although this work showed promising results, UV light as a trigger is not practical as it poses an adverse effect on the cell and tissue and has low tissue penetrability [200]. Visible light has also been reported as a trigger to release the cargo from MSNs. One work reported on the usage of the ruthenium complex as the gate to cap the MSNs’ pores. The result indicated that in the absence of light, the carrier was stable, and when Vis light was irradiated, the rapidly released cargo was observed. Interestingly, the gate, the ruthenium complex, can be controlled. Through heating at elevated temperatures, the pores re-closed, which can be re-opened upon irradiation [187]. Visible light can overcome the problem posed by UV, but it still has poor tissue penetration, which makes it difficult to be applied in cancer treatment. The best light wavelength to achieve good tissue penetration lies between 650–900 nm, which is in the NIR range. Therefore, the majority of light-responsive MSNs developed with NIR trigger [200]. One work reported on Janus gold nanostar-MSNs as NIR responsive carrier. The gold surface was functionalized with a thiolated photolabile molecule to bind itself to MSNs and β-cyclodextrin as the gate. Through NIR irradiation, the photolabile molecule dissociated and form succinic acid and this succinic acid induced the opening of the gate [189]. In another work, upconversion nanoparticles (UCNP) were coated with MSNs layer, and the gate employed was β-cyclodextrin. The β-cyclodextrin was linked to the MSNs layer with photo-cleavable pyrenemethyl ester. The principle of this light-responsive system as follows: 1) NIR light was irradiated to the carrier. Due to the presence of UCNP, the NIR was converted to Vis and UV light. 2) The pyrenemethyl ester bond was broken with higher energy light, and the β-cyclodextrin was dissociated, which led to the drug released [190].

Temperature is another stimulus being used to develop stimulus responsive MSNs. The important criteria in producing thermo-responsive MSNs are it should be stable during the circulation (37 °C) and the drug released at the locally heated tumor (~40–45 °C) [201]. One work reported on the production of thermo-responsive MSNs by incorporating a supported lipid bilayer (SBL) on the MSNs surface. This SBL also served as the gate to close the MSNs pores and trap the drug. At elevated temperature, the lipid bilayer became flexible and exhibited a liquid state. This caused it to be more permeable, which led to the drug released [193]. Besides that, the thermo-responsive polymer was used to coat the MSNs surface to develop thermo-responsive MSNs. Copolymer consisting of poly(N-isopropylacrylamine) and poly(methacrylic acid) (PNINAM-co-PMAA) was used as the coating of MSNs surface to trap the drug, doxorubicin. This copolymer served as both the gate and thermo-responsive component of the carrier. At 37 °C, which is lower than the low critical solution temperature (LCST), the copolymer swelled and blocked the MSNs pores. As the temperature increased (>LCST), the copolymer collapsed, and the drug was released [194]. Overall, through our literature search, we discovered that the articles on MSNs carriers that depend solely on temperature stimulus are lacking. Most articles report on the production of dual or more stimulus-responsive MSNs, which consists of temperature and any other stimulus.

#### 3.3.2. Multiple Stimulus

In recent years, we discovered that the production of stimulus-responsive-MSNs as a drug delivery agent is no longer limited to single stimuli. Most articles reported on the utilization of dual-responsive systems, and some even reported up to three responsive systems. The most common stimulus being used simultaneously is pH and redox stimulus. Lu and colleague reported on the usage of hollow MONs with disulfide bridge in the silica walls and supramolecular interaction between α-cyclodextrin and anilino alkane group as the gate to produce pH and redox responsive carrier. The results showed that at low pH (mimicking the tumor environment), the percentage of doxorubicin released was about 58%. This was reported due to the dissociation of α-cyclodextrin from protonated aniline. As state previously, the disulfide bond is susceptible to GSH, which present at a high concentration in cancerous cells. GSH will cleave the disulfide bond resulted in the breaking of hollow MONs walls, and the drug was released [114]. Through our literature search, there were three articles reported on the utilization of chitosan and disulfide bond to create pH and redox responsive MSNs. Chitosan is a pH-responsive biopolymer. In acidic conditions, chitosan may disassemble and lead to the pore opening and drug being released. By using a disulfide bond to link the MSNs and chitosan, a redox responsive system was created. With a high concentration of GSH, the disulfide bond was broken, which lead to chitosan gate opening and release of the drug [202,203,204]. Besides simultaneous use of pH and redox stimulus, there have been reports on pH and ROS stimulus being used. Interestingly, Song et al. reported that polydopamine served as both the pH and ROS responsive component of the carrier. At low pH, the amine group of polydopamine was protonated, which broke the interaction between the polymer and curcumin (drug). In the presence of hydrogen peroxide (ROS stimulus), the hydrogen bond between polydopamine and curcumin was broken, and the drug was released [205]. Dual responsive MSNs that are sensitive towards pH and temperature has also been developed. MSNs consist of carbon dots and poly(N-vinylcaprolactam) was produced as a pH and temperature-responsive carrier. The carbon dots and poly(N-vinylcaprolactam) were functionalized to the MSNs surfaces using a Schiff base bond. This Schiff base was reported to be acid sensitive and broken at acid atmosphere, which led to drug released. The polymer, poly(N-vinylcaprolactam), which served as a temperature-responsive molecule were at an elevated temperature; the polymer underwent phase transition and collapsed where the drug was then released from the carrier [206]. There have been other dual responsive systems reported and are summarized in Table 3.

Besides dual responsive systems, there have been reports on tiple responsive systems. pH and redox the common stimuli being used while the third stimulus varied. Chen and coworkers reported on triple responsive carriers, which were sensitive to pH, redox and enzyme stimuli. The MSNs were functionalized with gold via a sulfide bond and hyaluronic acid via an amide bond. The gold–sulfur bond, amide bond and hyaluronic acid were reported to be responsive to the redox, pH and enzyme stimulus, respectively [207]. In other work, a triply responsive system was developed to be sensitive towards pH, redox and temperature. The carrier consisted of multiwalled carbon nanotubes covered with mesoporous silica graft poly(*N*-isopropylacrylamide-*block*-poly(2-(4-formylbenzoyloxy) ethyl methacrylate) via disulfide linkages. As stated previously, the disulfide link is receptive towards redox stimulus. The drug was bonded to the polymer via Schiff base bond, and this bond was broken in acidic condition, which led to doxorubicin released. At elevated temperatures, the polymer collapsed. Thus, the drug was released [208]. Other articles related to triple responsive MSNs are summarized in Table 3.

### 3.4. Multidrug Carrier

Chemotherapy is a systemic treatment that poses a therapeutic effect on tumors with a tendency to metastasized to advanced tumors. However, there are several issues that need to be overcome, such as (1) Adverse side effects, (2) Most chemotherapeutic drugs have low aqueous solubility poor stability, (3) Poor delivery to achieve good efficacy and (4) Multidrug resistance (MDR). In a typical chemotherapy process, multiple drugs administered sequentially to increase treatment efficacy and delay [228]. An alternative to overcome these issues is by incorporating two or more drugs in a single carrier. They have been several reports that utilized MSNs as a carrier to deliver two anticancer drugs for treatment. Zhang and colleagues reported on the usage of hollow MONs to deliver doxorubicin and cisplatin drugs. The results showed the with two drugs; the anticancer activity was enhanced [119]. For the treatment of acute promyelocytic leukemia, MSNs were coated with folic acid-modified PEGylated lipid bilayer membrane to carrier paclitaxel and tanshinone IIA drugs [154]. Specifically, to overcome the MDR of cancer stem cells, MSNs were used to deliver doxorubicin and tariquidar [229]. MSNs carrier consists of polydopamine, and upconversion nanoparticles in yolk-shell configuration were produced to deliver doxorubicin and hydroxycamptothecin. This carrier was able to deliver both hydrophilic (doxorubicin) and hydrophobic (hydroxycamptothecin), where MSNs were used for doxorubicin and polydopamine for hydroxycamptothecin housing, respectively [230]. Similarly, hydrophilic and hydrophobic drug, pemetrexed and ellagic acid, respectively, were loaded onto MSNs. The ellagic acid was encapsulated within the MSNs pores through electrostatic interaction while pemetrexed was chemically anchored to the lactoferrin shell which surrounds the MSNs [231]. Interestingly, Janus nanoparticles consist of gold and MSN with an additional β-cyclodextrin gate was successfully synthesized to deliver paclitaxel and doxorubicin. The β-cyclodextrin served as the carrier for paclitaxel, while MSNs cargo was doxorubicin [232]. Overall, MSNs application as a drug delivery agent is not limited to anticancer or chemotherapeutic drugs; it can also serve as a carrier for gene therapy, immunotherapy or photothermal therapy.

## 4. Multifunctional Drug Delivery Agent

The usage of MSNs in the field of cancer treatment is no longer limited to only as drug delivery agents of anticancer drugs. Many studies have reported MSNs can serve in other types of treatment and diagnosis of cancer. In recent work, MSNs served as both controlled released delivery and imaging agent, respectively. Luminescent MSNs were generated by incorporating aggregation-induced emission (AIE) molecule, 10-phenylphenothiazine, into it. It was then coated with a pH-sensitive polymer to generate a pH-responsive carrier [233]. Another interesting study reported by Mira and coworkers employed MSNs for ultrasound imaging and therapy. The MSNs were stabilized by gas that can initiate cavitation at low and high acoustic intensity. They reported that under low acoustic pressure, ultrasound imaging was produced, while at high acoustic pressure, the microbubbles collapsed, which produced shockwaves and ablate the nearby cells or transiently perforate their membranes [234]. Lee and colleagues reported on multifunctional MSNs as imaging agents and carriers for photothermal therapy of cancer. They encapsulated 1,1′dioctadecyl-3,3,3′,3′-tetrametylindotrucarbocyanine iodide (DIR) and coated the MSNs with PEG to increase the carrier stability. This DIR served as an imaging agent as it can be detected through fluorescence imaging, and it also served as an agent for photothermal therapy. It can absorb NIR irradiation and generate heat, which can kill the cancer cells [235]. Similarly, Wang et al. reported on the usage of MSNs containing fluorescein isothiocyanate (FITC) and molybdenum disulfide (MoS_2_) as imaging and carrier for photothermal therapy. Direct conjugation of MoS_2_ with FITC will lead to intensity quench of fluorescence. Therefore, by employing MSNs as a carrier for both of these, better imaging and treatment were obtained where the quenching was minimized. The results showed that MoS_2_ has excellent photothermal conversion efficiency, which makes them a good photothermal agent, while the FITC in combination with MSNs and MoS_2_ showed excellent fluorescence [106]. Another common inorganic material incorporated in MSNs to develop multifunctional carrier is iron oxide. One study reported on the development of envelop-type MSNs functionalized with folic acid and iron oxide as targeting and imaging agent, respectively, to deliver quercetin. The MSNs were further functionalized with a pH-sensitive polymer to develop a pH-responsive system [96].

## 5. Conclusions

The research on finding the best method to combat cancer is an ongoing and continuous process. The approach to this is either through finding a more effective drug or improvement of the carrier. We believed that drug effectiveness would be worthless without the best carrier. Therefore, it is important to find and improve the delivery agent to increase drug efficacy. MSNs is one of the carriers that can overcome the issues posed by the current treatment. It has many desirable properties such as high surface area, tunable particle size and morphology and easy surface functionalization. These properties can increase the drug loading, improvement of circulation time and development of multifunctional carrier. Although the potential of MSNs as anticancer drug delivery agents is high, the understanding of its behavior in the human body is still limited. It is also important for us to understand the biosafety issue related to implementing MSNs as a carrier, such as renal clearance, toxicity inside the human body, effectiveness of its delivery inside the human body. Therefore, a great deal of work remains to develop MSNs with the best properties to combat cancer. However, with time and improvement, we can overcome this.

## Figures and Tables

**Table 1:** Summary of technique in MSNs synthesis.

Techniques	Templates	Silica Source	Additional Information	References
Sol–gel	PEG (Mw: 3000 g/mol)	Sodium silicate	Spherical * SA 685 m^2^ g^−1^	[18]
	CTAC	TEOS	Spherical * PS 49.73 nm	[19]
	CuS NPs (hollow template) CTAB (pore template)	TEOS	* PS 33, 44 and 48 nm with TEOS amount of 170, 180 and 200 μL, respectively. * SA 648.4 m^2^ g^−1^	[20]
	CTAB	TEOS	Lychee like MSNs * SA 536.065 m^2^ g^−1^	[21]
	CTAC	TEOS	Spherical * PS 21–111 nm	[22]
Hydrothermal	CTAB	TEOS	Spherical * PS less than 50 nm * SA ranging from 440 to 93 m^2^ g^−1^	[23]
	CTAB	TEOS	Spherical * PS 230 nm * SA 133 m^2^	[24]
	CTAB	TEOS	* SA 270.4 m^2^/g	[25]
	Pluronic F-127 (nonionic surfactant)	TEOS	SBA-15 type MSNs * SA 1098 m^2^/g * PS (DLS) 284 nm	[26]
Green	-	H_2_SiF_6_	Spherical * SA of 341, 327, 346 m^2^/g	[27]
	CTAB	Banana peels ash (sodium silicate)	-	[28]
	Pluronic F127	Rice husk	Spherical * PS 34.4 nm	[29]
	Tannic acid	TEOS	Spherical * PS 200–250 nm * SA 630–740 m^2^/g	[30]
	C_16_-l-amino acid Histidine Proline tryptophan	TEOS APTES	Sphere * PS 130–270 nm * SA 239–678 m^2^/g	[31]

* PS: particle size, SA: surface area.

**Table 2:** Various stimulus-responsive-MSNs for controlled release.

Gatekeeper	Stimulus	Cargo/Drugs	Ref
Polyacrylic acid	pH	Umbelliferone	[150]
Poly-l-lysine	pH	Doxorubicin	[166]
Poly(tannic acid)	pH	Doxorubicin	[167]
Hyaluronic acid	pH	Doxorubicin	[157]
Polyethylene glycol and chitosan	pH	Doxorubicin	[168]
Zinc oxide	pH	Doxorubicin	[169]
Albumin	pH	Lamivudine	[170]
Ferritin	pH	Doxorubicin	[162]
Beta-cyclodextrin	pH	5-fluorouracil	[171]
Chitosan	Redox	Rhodamine 6G	[172]
Cucurbit[6]uril, cyclopentyl methylamine and polyacrylic acid	Redox	Doxorubicin	[173]
Gold	Redox	Doxorubicin	[90]
Zinc sulfide	Redox	Doxorubicin	[174]
Ferrocene-containing amphiphilic block copolymer	Redox	Doxorubicin	[175]
Organosilica	Redox	Doxorubicin	[176]
Graphene	Redox	Rhodamine B	[177]
Bovine serum albumin	Redox	Epirubicin	[178]
Chitosan	Enzyme	Doxorubicin	[179]
Peptide	Enzyme	Organotin	[180]
Guar gum	Enzyme	5-fluorouracil	[181]
Hyaluronic acid	Enzyme	5-fluorouracil	[182]
Hyaluronic acid	Enzyme	Doxorubicin	[183]
Hyaluronic acid and collagen I	Enzyme	Doxorubicin	[184]
Iron oxide	Enzyme	Doxorubicin	[185]
Gold	Enzyme	Doxorubicin	[186]
Ruthenium complex	Light	Safranin O	[187]
α-cyclodextrin	Light	Doxorubicin	[188]
β-cyclodextrin	Light	Doxorubicin	[189]
β-cyclodextrin	Light	Camptothecin	[190]
β-cyclodextrin and -diazo-1.2-napthoquinones	Light	Doxorubicin	[191]
Human serum albumin	Light	Doxorubicin	[192]
Supported lipid bilayer	Temperature	Doxorubicin	[193]
Poly(N-isopropylacrylamine)-co-poly(methacrylic acid) (PNINAM-co-PMAA)	Temperature	Doxorubicin	[194]
Poly(N-isopropylacrylamide)-co-(1-butyl-3-vinyl imidazolium bromide) (p-NIBIm)	Temperature	Cytochrome C	[195]
PNINAM	Temperature	Methylene blue	[196]

**Table 3:** Multiple responsive MSNs with their responsive linker/moiety.

**Responsive linker/moiety**	**Stimulus**							**Ref**
	**pH**	**Redox**	**Enzyme**	**ROS**	**Temperature**	**Light**	**Other Compounds**	
	Dual stimulus							
	α-cyclodextrin and anilino alkane	Disulfide bond						[114]
	Polydopamine	Disulfide bond						[209]
	Chitosan	Disulfide bond						[210]
	Citraconic	Disulfide bond						[211]
	Chitosan	Disulfide bond						[202,203,204]
	Benzoic imine bonds	Disulfide bond						[158]
	Zinc oxide quantum dots	Disulfide bond						[212]
	Bull serum albumin	Disulfide bond						[213]
	Sodium alginate	Disulfide bond						[214]
	Polydopamine			Polydopamine				[205]
	Carboxymethyl chitin			Thioketal bond				[215]
	Schiff base bonds				Poly(N-vinylcaprolactam)			[206]
	PEG-like Jeffamine M-2005				Polyphosphazene			[216]
	Poly(N-isopropylacrylamide-co-methacrylic acid)				Poly(N-isopropylacrylamide-co-methacrylic acid)			[217]
	Polydopamine					Gold		[139]
	Hydroxyapatite					Gold		[218]
	Carboxylic acid						Chloride ions (Salt)	[219]
		Disulfide bond	Cystine-dopamine					[220]
		Dithiodipropionic		Selenocysteine				[221]
		Disulfide bond			Poly(*γ*-benzyl-l-glutamate)			[222]
		Disulfide bond				Azobenzene/galactose-grafted polymer		[223]
				Ferrocene	Poly(N-isopropylacrylamide)			[224]
	Triple stimulus							
	Amide bond	Gold-sulfur bond	Hyaluronic acid					[207]
	Schiff base bond	Disulfide bond			poly(*N*-isopropylacrylamide-*block*-poly(2-(4-formylbenzoyloxy) ethyl methacrylate)			[208]
	Polydopamine	Disulfide bond				Polydopamine		[225]
	Electrostatic interaction	Disulfide bond				Carbon dots		[226]
	Ester bond	Disulfide bond					Molecular interaction (glucose)	[227]

## Data Availability

Data is contained within the article.
